# High expression of periostin is dramatically associated with metastatic potential and poor prognosis of patients with osteosarcoma

**DOI:** 10.1186/1477-7819-12-287

**Published:** 2014-09-15

**Authors:** Fei Hu, Wei Wang, Hang-Cheng Zhou, Xi-Fu Shang

**Affiliations:** Department of Orthopedics, Anhui Provincial Hospital, Anhui Medical University, 17# Lujiang Road, Hefei, 230001 People’s Republic of China; Department of Medical Oncology, Anhui Provincial Hospital, Anhui Medical University, 17# Lujiang Road, Hefei, 230001 People’s Republic of China; Department of Pathology, Anhui Provincial Hospital, Anhui Medical University, 17# Lujiang Road, Hefei, 230001 People’s Republic of China

**Keywords:** Periostin, Osteosarcoma, Prognosis, Metastasis

## Abstract

**Background:**

Recent studies have found that periostin (PN), as a kind of secreted glycoprotein, is closely related to the metastatic potential and prognosis of many kinds of tumors. This study aimed to examine the expression of PN in patients with osteosarcoma and explore the relationship of PN expression with clinicopathologic factors and prognosis.

**Methods:**

PN was detected by histopathological and immunohistochemical methods in 62 cases of osteosarcoma and 62 of osteochondroma. Detailed pathological and clinical data were collected by reviewing medical records.

**Results:**

The results showed that increased PN protein expression was prevalent in osteosarcoma and was significantly associated with pathologic subtype (*P* =0.000), tumor size (*P* =0.016) and Enneking stage (*P* =0.047). Additionally, expression of PN was found to be an independent prognostic factor in osteosarcoma patients. High expression of PN protein is closely correlated to the tumor progression and poor survival of osteosarcoma.

**Conclusions:**

Our data suggest that PN is a promising biomarker for identifying individuals with poor prognostic potential and suggests its possible use as a prognostic marker in patients with osteosarcoma.

## Background

Osteosarcoma accounts for approximately 20% of primary malignancies of bone, which is the most common malignant primary bone tumor among children and adolescents [1]. It frequently tends to develop distant metastasis and ultimately results in death, especially in cases of lung metastasis. About 20% of patients present visible metastases with imaging at diagnosis and a quarter patients have metastases during the course of treatment [2]. However, due to lack of effective and efficient tumor biomarkers for early diagnosis, and that any treatment for relapse is absolutely resistant to the relapsed tumor, the prognosis remains poor and most patients die at the advanced stages. Thus, developing a novel predictor for anticipating the invasive potential and prognosis of osteosarcoma is significantly crucial.

Periostin (PN), a member of the fasciclin domain and also called osteoblast-specific factor 2 (OSF-2), is a disulfide-linked cell adhesion protein that has been shown to be expressed preferentially in the periosteum and periodontal ligaments, acting as a critical regulator for bone and tooth formation and maintenance [3–6]. Recently, accumulating evidence has revealed that PN is overexpressed in various human cancers including liver, head and neck, neuroblastoma, breast, colon, esophageal, ovary, and so on [7–17]. Furthermore, PN as a promising marker for tumor aggression in different types of human cancer have been identified [8, 10]. In the past, although many studies have focused on the multiple facets of PN in bone metabolism [18–23], few were reported about the expression of PN in bone-related tumors and its potential clinical significance.

In the present study, we have examined the expression levels of PN in osteosarcoma and analyzed its correlation with clinicopathological characteristics and prognosis.

## Methods

### Patients and tissue samples

A total of 124 patients (62 cases with osteosarcoma and 62 with osteochondroma) who underwent surgical resection between 2003 and 2010 were selected from the Department of Orthopedics in Anhui Provincial Hospital (China). These patients with osteosarcoma were treated according to the standardized protocol consisting of neoadjuvant chemotherapy (four cycles of methotrexate and cisplatin were administered with a minimum of a 21-day interval between consecutive cycles), followed by appropriate surgical management and postoperative adjuvant chemotherapy. All tissue specimens were formalin-fixed and paraffin-embedded for immunohistochemistry staining. Detailed pathological and clinical data were collected by reviewing medical records, including age and gender, age at diagnosis, tumor size, site of primary disease, histologic subtype, complete blood count, erythrocyte sedimentation rate, alkaline phosphatase, serum albumin, and Enneking stage. A total of 62 patients with osteosarcoma were comprised of 33 males and 29 females, with the mean age of 25 ± 12-years-old (range: 6 to 52 years). Two pathologists who were blinded to the clinical information confirmed all histological diagnoses and judged the degree of staining independently.

All patients involved in this study had signed the informed consent. The study protocol conformed to the ethical guidelines of the Declaration of Helsinki. Ethical approval for the use of human subjects was obtained from the research ethics committee of Anhui Medical University.

Follow-up was terminated on 8 August 2013. The mean follow-up was of 36.6 months (range: 25 to 92 months). After surgical resection, all patients were monitored using X-ray as preferential, abdominal ultrasonography, technetium bone scans, chest computed tomography (CT) scans and/or magnetic resonance imaging (MRI) scans every three to six months, prospectively. Overall survival (OS) was defined as the interval between surgery and death or the last observation taken. The data were censored at the last follow-up period for living patients. Relapse-free survival (RFS) was defined as the interval between the date of surgery and the date of diagnosis of any type of relapse or the last follow-up assessment.

### Histopathological and immunohistochemical analyses

Immunohistochemistry was used to examine the expression of PN in all tissue specimens. Rabbit anti-periostin antibody was ordered from Beijing Biosynthesis Biotechnology Co., Ltd (Beijing, China). PV-6000 Power Vision ™ Two-step histostaining reagent, 3,3-diaminobenzidine tetrahydrochloride (DAB) and phosphate-buffered saline (PBS) were obtained from Beijing Zhongshan Golden Bridge Biotechnology Company (Beijing, China). The whole process was followed by the manufacturer’s instructions. Briefly, the tumor tissues were fixed in 10% formalin, embedded in paraffin and cut into 4-μm-thick sections. The sections were stained with hematoxylin and eosin (HE) for histological examination. The tissue sections were deparaffinized and rehydrated in a graded series of alcohols. The sections were microwaved in citrate buffer (pH 6.0) for 20 minutes and then cooled for an additional 20 minutes at room temperature. Endogenous peroxidase activity was blocked with 3% hydrogen peroxide for 10 minutes. The sections were then immunostained with PN antibody (1:50) (Zhongshan Jinqiao Co., Beijing, China) and incubated at 4°C overnight. After rinsing with PBS three times for 5 minutes each, the sections were incubated in horseradish peroxidase-conjugated secondary antibody (Zhongshan Jinqiao Co., Beijing, China) for 20 minutes. After being washed again, peroxidase activity was visualized with DAB as a chromogen. Sections were counterstained with hematoxylin, dehydrated and mounted. The negative controls were processed in a similar manner with PBS instead of primary antibody. The result of immunohistochemistry was expressed by the staining cells on a graduated percentage (0 to 100%). Staining of under 10% of the tumor cells showed weak or focal immunopositivity or no staining was clarified as negative., staining of 10 to 30% of tumor cells showed moderate or patchy immunopositivity as +, and staining of over 30% of tumor cells showed strong or diffuse immunopositivity as ++. A total of 10 fields were selected, and expression in 1,000 tumor cells (100 cells/field) was evaluated using a high-power (400×) microscope [9].

### Statistical analysis

All statistical analyses were performed using SPSS 13.0 (SPSS Inc., Chicago, Illinois, United States). χ^2^ and Fisher’s exact test was performed to assess associations between PN expression and clinicopathological parameters. The Kaplan-Meier method was used for survival analysis, and differences in survival were estimated using the log-rank test. A multivariate survival analysis was performed for all parameters that were significant in the univariate analysis using the Cox regression model. *P* <0.05 was considered to be statistically significant.

## Results

### Immunohistochemical expression of periostin in osteosarcoma and osteochondroma

**Figure 1:**
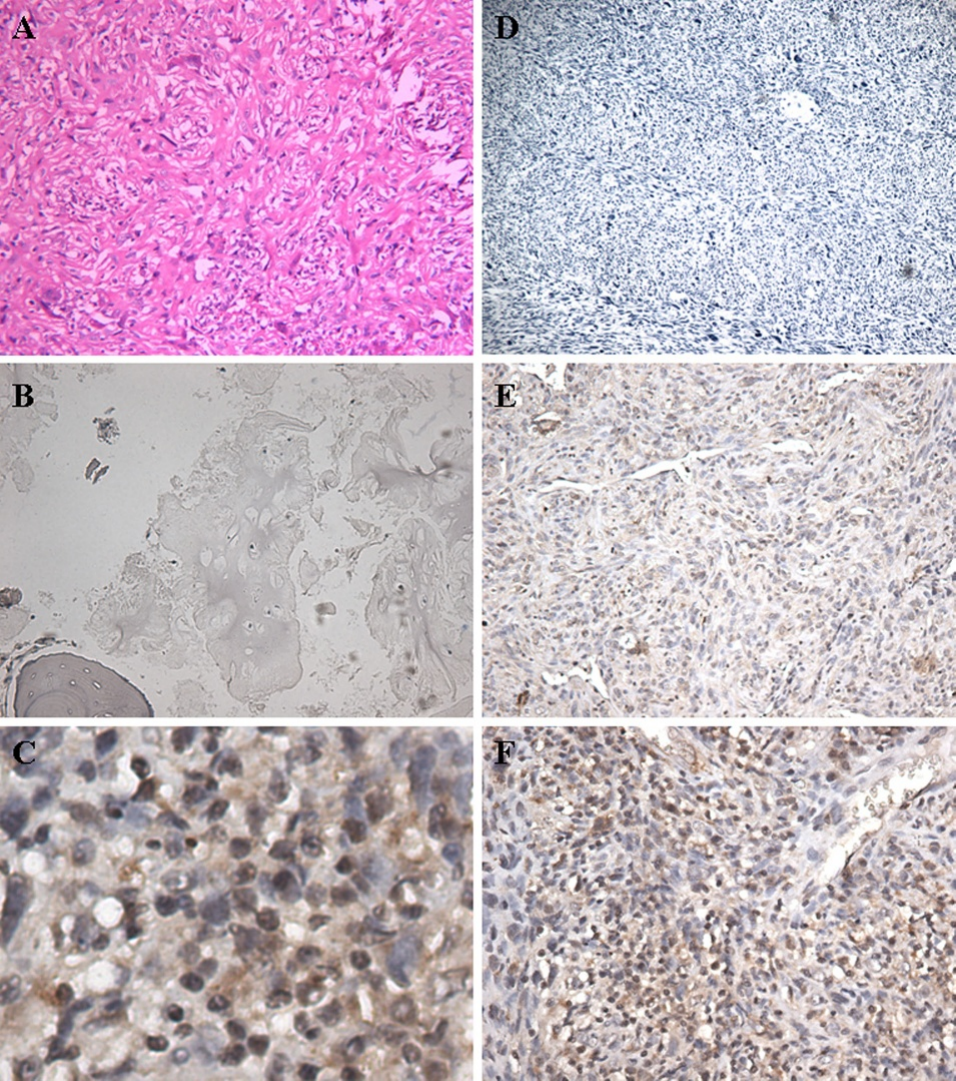
**Immunohistochemical staining of periostin in osteosarcoma and osteochondroma tissues.** Periostin (PN) mainly expressed in the cytoplasm of osteosarcoma tissues. **(A)** H&E staining of osteosarcoma, ×100; **(B)** negative for PN staining in osteochondroma, ×100; **(C)** ++ for PN staining in osteosarcoma, ×400; **(D)** negative for PN staining in osteosarcoma, ×40; **(E)** + for PN staining in osteosarcoma, ×100; **(F)** ++ for PN staining in osteosarcoma, ×100.

Immunohistochemical analysis revealed that the positive expression of PN was mainly localized in the cytoplasm, with some tumor cells stained strongly, while others exhibited little or no staining at all (Figure 1). The positive rates of PN in osteosarcoma tissues were 80.6% (50 out of 62) and 17.7% in osteochondroma (11 out of 62), and PN expression in osteochondroma was significantly lower than that in osteosarcoma tissues (Table 1, *P* <0.05).

**Table 1:** **Differential expression of periostin between osteosarcoma tissues and corresponding osteochondroma (124 cases)**

Tissues	Case number	Periostin		Positive rate (%)
		Positive	Negative	
Osteosarcoma	62	50	12	80.6%
Osteochondroma	62	11	51	17.7%

To elucidate its clinical significance we evaluated the association between PN expression and clinicopathological characteristics, including age, sex, location, pathologic subtype, tumor size, Enneking stage, alkaline phosphatase, serum albumin and erythrocyte sedimentation rate (ESR). A detailed histological subtype showed 57 cases of conventional osteosarcoma, 5 cases of special osteosarcoma (including 4 cases of low-grade central osteosarcoma) and 1 case of parosteal osteosarcoma. As shown in Table 2, the expression level of PN was associated with pathologic subtype (*P* = 0.000, odds ratio (OR) = 8.143), tumor size (*P* = 0.016, OR = 9.370) and Enneking stage (*P* = 0.047, OR = 1.324), but there was no significant correlation with age, gender, location, alkaline phosphatase level, serum albumin level and ESR.

**Table 2:** **Periostin expression status in relation to selected clinicopathologic features in 62 osteosarcoma patients (cases)**

Clinicopathologic data	Case number	Periostin		***χ*** ^***2***^	***P*** value	OR	95% CI
		Positive	Negative				
Sex							
Male	33	26	7	0.156	0.693	0.774	0.216 ~ 2.768
Female	29	24	5				
Age at diagnosis (years)							
<30	42	34	8	0.008	0.929	0.941	0.247 ~ 3.592
>30	20	16	4				
Site of primary disease							
Tibia	26	22	4	0.995	0.802		
Femur	16	13	3				
Humerus	10	7	3				
Other	10	8	2				
Histologic subtype							
Special	5	0	5	22.661	0.000	8.143	4.069 ~ 16.297
conventional	57	50	7				
Enneking stage							
I, II	49	37	12	3.948	0.047	1.324	1.129 ~ 1.553
III	13	13	0				
Size (at diagnosis) (cm)							
<5	38	27	11	5.787	0.016	9.370	1.123 ~ 78.169
>5	24	23	1				
Alkaline phosphatase (u/L)							
<500	35	28	7	0.021	0.884	0.909	0.254 ~ 3.258
>500	27	22	5				
Serum albumin (g/L)							
<30	22	18	4	0.03	0.862	0.889	0.235 ~ 3.367
>30	40	32	8				
ESR (mm/hr)							
<30	45	36	9	0.044	0.834	0.857	0.202 ~ 3.636
>30	17	14	3				

CI: Confidence interval; ESR: erythrocyte sedimentation rate; OR: the odds ratio; PN: Periostin.

*P* <0.05 was considered to be statistically significant.

### Relationship between PN expression and prognosis in osteosarcoma

**Figure 2:**
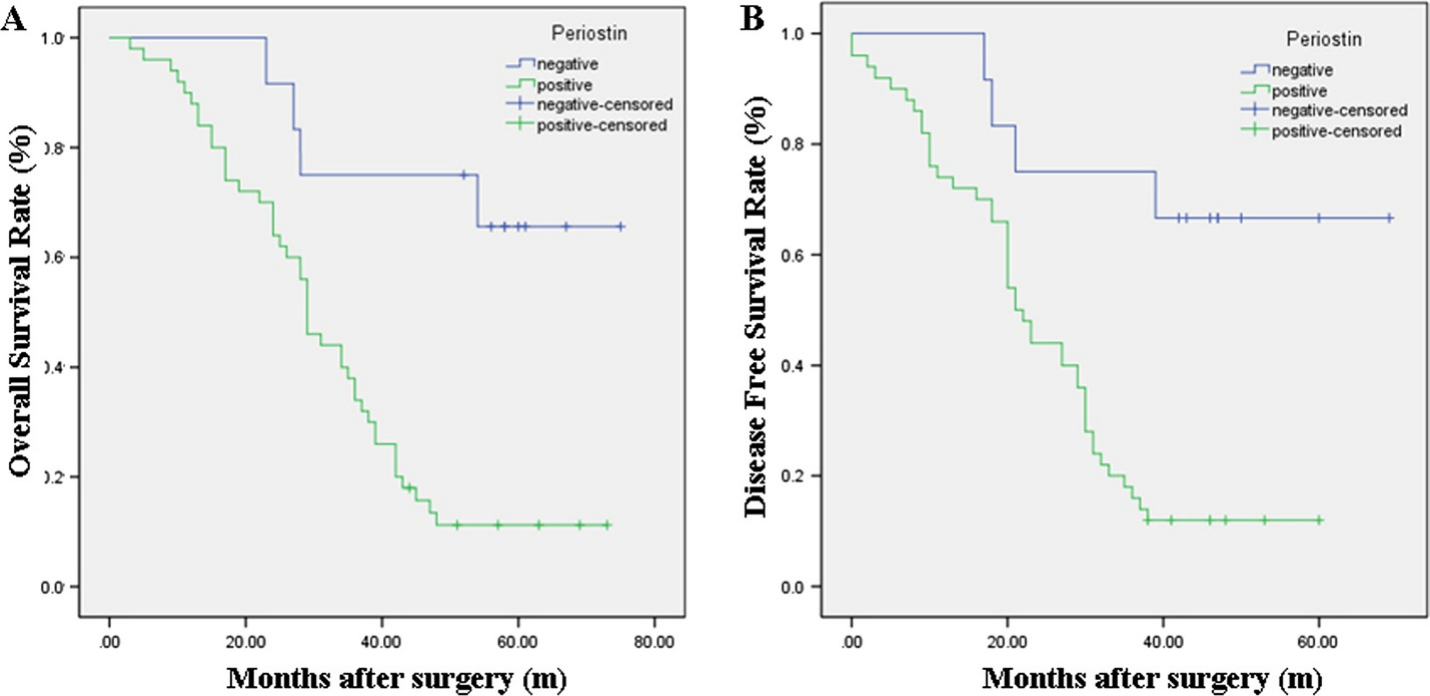
**Kaplan-Meier analysis of overall survival (OS) and disease-free survival (DFS) curves of patients with osteosarcoma based on periostin expression as positive or negative. (A)** OS curve of patients with osteosarcoma based on periostin expression; **(B)** DFS curve of patients with osteosarcoma based on periostin expression. The osteosarcoma patients with periostin-positive expression showed significantly poorer OS and DFS rates than those with periostin-negative expression.

**Figure 3:**
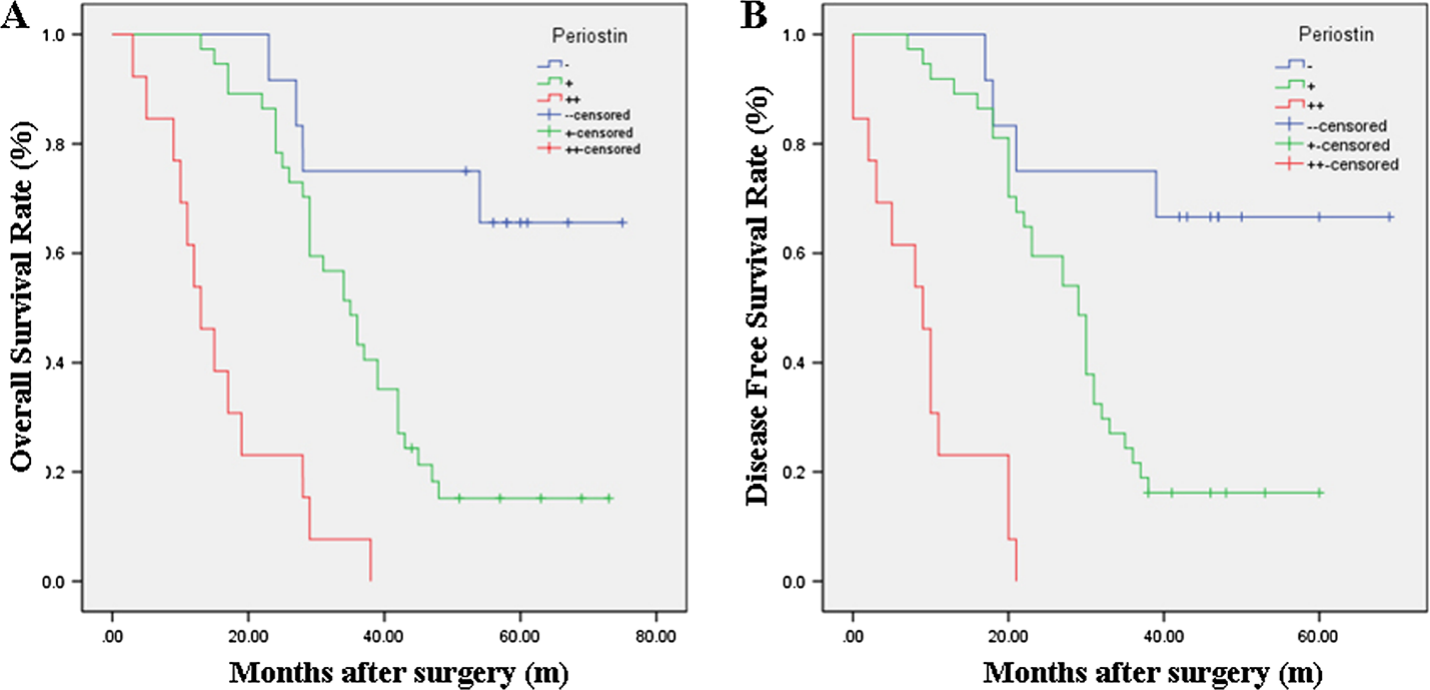
**Kaplan-Meier analysis of overall survival (OS) and disease-free survival (DFS) curves of patients with osteosarcoma based on periostin expression as strongly positive, weakly positive or negative. (A)** OS curve of patients with osteosarcoma based on periostin expression; **(B)** DFS curve of patients with osteosarcoma based on periostin expression. The osteosarcoma patients with periostin-positive expression showed significantly poorer OS and DFS rates than those with periostin-negative expression. The survival of patients in the strongly positive periostin expression was poorest.

Patients with PN-positive expression showed a poorer prognosis than those with PN-negative expression by the Kaplan-Meier analysis. The log-rank test revealed that the overall survival time of osteosarcoma patients with PN-positive expression was markedly shorter than that with PN-negative expression (*P* = 0.003; Figure 2A; Table 3). Furthermore, similar results were also observed in the disease-free survival analysis (*P* = 0.001; Figure 2B; Table 3). To understand deeply about the relationship between the PN-positive expression and prognosis, two different degrees of positive expression were analyzed using the Kaplan-Meier method (Figure 3). Moreover, as seen in Table 4, multivariate Cox analysis indicated that PN expression was one of the independent prognostic factors, along with pathologic subtype, Enneking stage and tumor size. At the last follow-up appointment, 44 patients in the PN-positive groups had died, with a median survival time of 31 months. Of these, two patients developed local recurrence and three had lung metastasis. Comparatively, six patients died in the PN-negative groups and the median survival time was 51 months.

**Table 3:** **Univariate analysis of factors associated with OS and DFS**

Variable	OS			DFS		
	Mean survival month	95% CI	***P*** value	Mean survival month	95% CI	***P*** value
Periostin						
Negative	51	42.191-60.975	0.003	42	32.503-50.663	0.001
Positive	31	26.533-35.347		23	19.578-29.222	
Sex						
Male	34	30.480-39.391	0.073	26	22.971-30.868	0.066
Female	31	25.388-36.612		23	16.055-29.945	
Age at diagnosis (years)						
<30	35	30.480-39.391	0.169	26	22.971-30.868	0.169
>30	31	25.388-36.612		23	16.055-29.945	
Site of primary disease						
Tibia	31	25.388-36.612	0.172	26	22.971-30.868	0.176
Femur	31	25.042-38.833		25	18.766-31.234	
Humerus	35	19.171-53.424		28	12.358-42.842	
Other	41	36.846-45.617		32	28.577-35.500	
Histologic subtype (n)						
Special	67	60.383-74.817	0.000	56	46.412-65.988	0.000
Conventional	32	28.044-36.096		24	20.849-27.852	
Enneking stage						
I, II	40	35.700-44.668	0.000	31	27.668-35.556	0.000
III	15	10.727-19.580		9	5.144-13.318	
Size (at diagnosis) (cm)						
< 5	44	39.965-49.193	0.000	35	31.469-39.636	0.000
> 5	19	15.395-23.938		13	9.586-16.914	
Alkaline phosphatase (u/L)						
< 500	33	30.480-38.391	0.162	26	22.971-30.868	0.151
> 500	31	25.388-36.612		23	16.055-29.945	
Serum albumin (g/L)						
< 30	35	31.224-39.031	0.211	27	22.845-31.547	0.278
> 30	31	25.308-35.578		24	18.454-28.567	
ESR (mm/hr)						
< 30	34	31.135-39.023	0.060	26	21.975-30.086	0.126
> 30	31	25.886-34.479		23	19.333-28.792	

CI: Confidence interval; DFS: disease free survival; ESR: erythrocyte sedimentation rate; OS: Overall survival;

*P* <0.05 was considered to be statistically significant.

**Table 4:** **Multivariate analysis of factors associated with OS and DFS**

Variable	OS			DFS		
	Hazard ratio	95% CI	***P*** value	Hazard ratio	95% CI	***P*** value
Periostin (Negative versus positive)	3.751	1.564-8.367	0.001	3.403	1.644-7.908	0.001
Pathologic subtype (Conventional versus special)	2.434	1.275-4.564	0.003	2.822	1.577-5.057	0.001
Enneking stage (I, II versus III)	3.156	1.788-5.693	0.000	3.172	1.809-5.629	0.000
Tumor size, cm (≤5 versus >5)	2.039	1.108-3.784	0.021	2.024	1.137-3.70	0.018

CI: Confidence interval; DFS: disease free survival; OS: Overall survival. P <0.05 was considered statistically significant.

## Discussion

Periostin, originally named as osteoblast-specific factor-2 (OSF-2) and first identified in bone, has been implicated in regulating adhesion and differentiation of osteoblasts [3–5]. The molecular structure of PN is particularly highly homologous to ßig-h3, which promotes cell adhesion and the spreading of fibroblasts [3]. By RNA dot-blot analysis, PN expression was observed in a wide range of normal adult tissues, including aorta, stomach, lower gastrointestinal tract, placenta, uterus and breast [24]. Recently, it has been frequently reported that PN is overexpressed in various types of human malignant tumors. For instance, Kudo *et al*. [9] reported PN was overexpressed in oral cancer cells and enhanced migration and invasion. Furthermore, *in vitro* studies such as Bao *et al*. [14], demonstrated that highly vascular metastatic tumors derived from the periostin-producing cells showed fewer apoptotic cells than control cells. Accumulated findings indicated that overexpression of PN might be common in tumor development. However, some authors have reported that downregulation of PN mRNA was significantly related to higher grade bladder cancer [25, 26]. Furthermore, Kanno *et al*. [27] have demonstrated that PN has biphasic effects on the migration of pancreatic carcinoma. These varied findings suggest that PN might have different functions for different pathological types of cancer. However, PN expression status and the relation with prognosis in osteosarcoma have not been clearly explained until now.

Osteosarcoma is the most common malignant primary bone tumor with a high capacity for distant metastasis. Although the survival rate for patients with osteosarcoma has significantly improved over the past two decades through the use of a combination of aggressive chemotherapy and surgery, metastatic or recurrent disease still occurs in 30 to 40% of these patients and the majority of those succumb to the disease [28]. Therefore, it is critical to identify the tumor metastasis-associated biomarkers of osteosarcoma. In the present study, we discovered that the PN expression in tumor tissues of patients with osteosarcoma was significantly increased compared with those with osteochondroma. Furthermore, we found that osteosarcoma with PN-positive expression were more frequently Enneking stage III, with a tumor size >5 cm, and a conventional pathologic subtype than osteosarcoma with PN-negative expression. Accumulated findings indicated that OS and DFS were better in patients without PN expression than those in patients with PN-positive expression. Both Kaplan-Meier and multivariate analysis showed that the expression of PN was an independent predictor of poor prognosis for both OS and DFS. The relationship between PN staining intensity and patient survival was also analyzed, and a general inverse trend between a decline in patient survival and an increase in PN staining intensity was observed. As observed in other cancers, increased expression of PN was associated with cell proliferation, adhesion, and migration. These results indicated that PN expression had an adverse influence on the osteosarcoma outcome. PN may be used as a marker to predict osteosarcoma patients’ prognosis.

A major limitation of this single-center study is its relatively small sample size. Secondly, the underlying molecular mechanisms are not been explored in this study. Recently, some studies revealed that PN binding to the integrins activates the focal adhesion kinase (FAK)- and protein kinase B (Akt/PKB)-mediated signaling pathways which promote tumor angiogenesis, invasion, and metastasis [29, 30]. On the other hand, Windischhofer *et al*. [31] demonstrated that PN was abundantly expressed in human MG-63 osteosarcoma cells. Stimulation of cells with Lysophosphatidic acid (LPA) resulted in a decrease of PN that was strictly dependent on early growth response-1 (Egr-1) expression levels mediated strictly via the Gαi/Src/p42/44 MAPK(mitogen-activated protein kinases) pathway with no involvement of the Gαq/11/PLC/PKC (phospholipase C, protein kinase C) or the PLD (phospholipase D)/PI3 kinase/Akt pathways. Thus, to illuminate these findings, further investigations will still be required to explore its exact mechanisms.

## Conclusions

In conclusion, our study found that PN expression was higher in osteosarcoma tissues compared with osteochondroma tissues and its overexpression was closely correlated with Enneking stage, tumor size, and pathologic subtype. Taken together, these preliminary results suggest that the overexpression of PN is a potential prognostic factor for osteosarcoma development and progression. In future, if this activity could be blocked by some specific inhibitors, it could provide a new target for the treatment of osteosarcoma.

## Abbreviations

DAB: 3,3-diaminobenzidine tetrahydrochloride
Egr-1: early growth response-1
FAK: focal adhesion kinase
HE: Hematoxylin and eosin
LPA: Lysophosphatidic acid
MAPK: mitogen-activated protein kinases
OS: Overall survival
OSF-2: Osteoblast-specific factor 2
PBS: Phosphate-buffered saline
PKB: protein kinase B
PKC: phospholipase C
PLD: phospholipase D
PLC: phospholipase C
PN: Periostin
RFS: Relapse-free survival.
